# Development of the Proactive Behavior Scale for Mid-Career Nurses: a reliability and validity study

**DOI:** 10.20407/fmj.2024-015

**Published:** 2024-10-31

**Authors:** Satomi Koyama, Yumiko Miyoshi, Kimie Takehara

**Affiliations:** 1 Graduate School of Health Sciences, Fujita Health University, Toyoake, Aichi, Japan; 2 Faculty of Nursing, Fujita Health University, School of Health Sciences, Toyoake, Aichi, Japan

**Keywords:** Mid-career nurses, Proactive behavior, Organizational socialization, Scale development

## Abstract

**Objectives:**

This study aimed to assess the reliability and validity of the Proactive Behavior Scale for Mid-Career Nurses, which was designed to measure the proactive behavior of nurses who have worked outside their current employment hospital (mid-career nurses) and who are in the process of organizational socialization.

**Methods:**

A mail survey was administered to 875 mid-career nurses using a draft version of the Proactive Behavior Scale. An exploratory factor analysis was conducted on the data, and a Proactive Behavior Scale for Mid-Career Nurses was developed (Survey 1). Additionally, a mail survey was administered to 706 mid-career nurses to examine the reliability and validity of the scale (Survey 2).

**Results:**

From the exploratory factor analysis results, three factors and 21 items were extracted. The three factors were (1) positive actions toward self-reliance, (2) positive actions toward reliable nursing practices, and (3) positive actions toward building relationships. The Cronbach’s alpha coefficient for the overall scale was 0.89, Spearman’s rank correlation coefficient for the test-retest method was 0.75, and Pearson’s product–moment correlation coefficient for the external criterion, the Scale of Proactive Behavior in Organizational Socialization for New Graduate Nurses and the Scale of Organizational Resocialization were 0.72 and 0.40, respectively.

**Conclusions:**

The Proactive Behavior Scale for Mid-Career Nurses has good reliability and validity.

## Introduction

To provide quality nursing care, each nurse should be aware of the philosophy, values, and behavioral patterns of their hospital’s organization, and practice nursing accordingly. Nurses frequently leave their jobs, with over 100,000 nurses re-entering the workforce each year in Japan.^1,2^ It is expected that organizational culture and behavioral patterns will differ from hospital to hospital, and that there will be differences in nursing practice methods. Therefore, even with experience and practical skills, it is difficult for mid-career nurses to practice nursing based on ideas and behaviors that fit the new organization immediately after re-entering the workforce. The inability to fully demonstrate one’s inherent ability to practice nursing is a significant loss. Therefore, to guarantee the quality of nursing care, mid-career nurses must adapt quickly to new organizations.

The process during which individuals adapt to a new organization is known as organizational socialization.^3^ Organizational socialization is a learning process, facilitated by the accomplishment of learning tasks such as cultural challenges to accept organizational norms and behavioral patterns, role challenges to perform the role, and technical challenges to acquire the skills necessary to perform the role.^4,5^ Achieving these learning tasks is facilitated by both support from the organization and the effort of the mid-career nurses themselves. Support from the organization is referred to as socialization tactics and includes on-the-job and newcomer training. In contrast, effort by mid-career nurses themselves is referred to as proactive behavior. Ogawa defines proactive behavior as “the overall proactive behavior of an individual to acquire the social knowledge and skills necessary to assume a role in an organization.”^6^ Proactive behavior has been reported to influence socialization learning and promote organizational socialization, leading to workplace adaptation.^7^ Konishi identified acquiring the necessary knowledge and skills in a new department, attitude of acceptance of the norms and values of the new department, behaviors that contribute to the new department by using experience, and understanding of the norms of the new organization as learning tasks in the organizational socialization of mid-career nurses.^8^ Therefore, in mid-career nurses’ organizational adaptation, proactive behavior by mid-career nurses themselves (proactive actions taken by them in the process of organizational socialization to adapt to the organization) toward the achievement of these learning tasks is important.

Research on proactive behavior in the nursing field in Japan is scarce, with the only study by Ukawa focusing on new graduate nurses, with no mention of mid-career nurses.^9^ Therefore, the authors developed a scale to measure the proactive behavior exhibited by mid-career nurses during organizational socialization. The development of the scale will enable us to identify proactive behaviors that are effective for organizational adaptation and discuss support for encouraging these behaviors. To conduct this study, a draft Scale of Proactive Behaviors for Mid-career Nurses comprising 60 items was developed beforehand. The draft scale was developed based on interviews with mid-career nurses, and the content validity of each draft item was confirmed. This study aimed to conduct a survey using the draft scale to develop the Proactive Behavior Scale for Mid-Career Nurses and assess the reliability and validity thereof.

## Methods

### Definition of terms

#### Mid-career nurses

This study defined a mid-career nurse as “nurses who have worked at other hospitals than the one where they are currently employed.”

#### Proactive behavior

This study defined proactive behavior as “proactive actions taken by mid-career nurses themselves in the process of organizational socialization to adapt to the organization.”

### Survey 1: Creating the Proactive Behavior Scale for Mid-Career Nurses

#### Participants

A list of 7,661 hospitals in Japan, stratified by 100 beds, was created based on a list of healthcare institutions published on the website of each local health (branch) bureau. The number of hospitals requested was determined by setting the target number of respondents to collect the survey to approximately 500, considering the percentage of hospitals that would cooperate (approximately 30%) and the collection rate of the survey (approximately 30%). A random number table was generated for each list classified by the number of beds, and proportionate stratified random sampling was used to select the target hospitals for the survey request. We requested a survey from the heads of nursing officers of 618 hospitals, and 875 mid-career nurses working at 97 hospitals who gave their consent were included.

#### Survey content

The survey comprised 60 draft items on a Proactive Behavior Scale for Mid-Career Nurses and basic attributes. The response format for the scale items was a Likert scale, with scores ranging from 7 (very applicable) to 1 (not applicable) for each item.^10^

#### Data collection method

An anonymous questionnaire survey was conducted via mail between May and August 2022. Written requests for the number of participants, questionnaires, and return envelopes were sent to the chief nursing officers of the hospitals that cooperated in the survey, and each participant was asked to distribute them individually. The survey was returned to the researcher by the participants with a self-addressed envelope.

#### Analysis method

After excluding missing values and outliers (values exceeding 1.5 times the upper and lower quartile ranges both above and below) to determine the analysis, the distribution of scores for each item was checked for ceiling and floor effects, kurtosis and skewness, and histograms. In addition, we checked inter-item correlations and Corrected Item-Total Correlation (CITC). An exploratory factor analysis was conducted using data that had undergone item analysis, and a scale was created. IBM SPSS Statistics 27 was used for statistical analysis.

### Survey 2: Examination of criterion-related validity and the test-retest method reliability

#### Participants

As in Survey 1, 619 hospitals different from those in Survey 1 were selected from a generated list of hospitals by proportionally stratified random sampling and requested to survey their head of nursing officers. Of these, 706 mid-career nurses working at 88 hospitals who provided consent were included in this survey.

#### Survey content

The survey consisted of the Proactive Behavior Scale for Mid-Career Nurses developed in Survey 1, the Scale of Proactive Behaviors in Organizational Socialization of New Graduate Nurses, the Scale of Organizational Resocialization, and basic attributes.

##### Scale of proactive behaviors in organizational socialization of new graduate nurses

Ukawa’s Scale of Proactive Behaviors in Organizational Socialization of New Graduate Nurses is a five-point Likert scale comprising 20 items with four factors: five items for behavior to master nursing skills, four items for human relationship forming behavior, six items for active learning behavior, and five items for behavior exploring feedback from others.^9^ This scale was adopted as an external criterion because it measures proactive behaviors that new nurses engage in during the process of organizational socialization and is predicted to be related to proactive behaviors in the organizational socialization of mid-career nurses, who are also in the nursing profession.

##### Scale of organizational resocialization

Nakahara’s Scale of Organizational Resocialization is a five-point Likert scale comprising four factors and 19 items: seven items for networking political knowledge acquisition, four items for learning rejection, five items for evaluation criteria and role acquisition, and three items for skills and knowledge acquisition.^5^ The scale is based on a survey conducted with mid-career sales employees and measures the degree of organizational socialization. Proactive behavior was adopted as an external criterion because it is predicted to be related to organizational socialization and is considered a facilitator of organizational socialization.

#### Data collection method

An anonymous questionnaire survey was conducted via mail between September and December 2023. To confirm the stability of the scale, the same participants were surveyed only on the Proactive Behavior Scale for Mid-career Nurses after an interval of approximately two to four weeks (the test-retest method). A request document, two questionnaires, and a return envelope were sent to the head of nursing officer regarding the number of participants and were distributed to each participant.

To confirm the stability of the scale, the same participants were surveyed only on the Proactive Behavior Scale for Mid-career Nurses after an interval of approximately two to four weeks (test-retest method). The questionnaires were individually returned to the researcher by the participants.

#### Analysis method

To examine criterion-related validity, Pearson’s product–moment correlation coefficient was calculated after checking whether each scale score was normally distributed. The test-retest method was used to confirm scale stability. After checking whether the first and second scores were normally distributed, Spearman’s rank correlation coefficient was calculated. IBM SPSS Statistics 27 was used for the statistical analysis.

#### Ethical considerations

The participants were informed of the study purpose and methods, protection of their personal information, and voluntary participation in the study. They confirmed their willingness to cooperate by responding to a questionnaire. This study was approved by the Medical Research Ethics Review Committee of Fujita Health University (Reception No.: HM20-609).

## Results

### Survey 1: Creation of the Proactive Behavior Scale for Mid-Career Nurses

#### Background of participants

Responses were received from 506 participants (57.8% response rate). Of these, 32 respondents with missing values, outliers, etc. and 107 respondents who indicated that they had or were unsure of their intention to retire were excluded, leaving a total of 367 respondents for analysis. Participants’ backgrounds are listed in Table 1.

#### Exploratory factor analysis

The histograms confirmed that the distribution of scores for each item was unimodal and not highly skewed. The kurtosis and skewness,^11^ ceiling and floor effects,^12^ CITC,^13^ and Cronbach’s alpha coefficient when items were deleted were checked; 11 items that did not meet the criteria were excluded. Ultimately, an exploratory factor analysis was conducted on 49 items.

Principal factor analysis and promax rotation were performed. The Kaiser–Meyer–Olkin sample validity was 0.94, which was above the criterion of 0.50. The Bartlett’s sphericity test p-value was less than 0.001, indicating that the covariance was not zero and that there was a correlation among the independent variables. These results confirmed that the data were acceptable for the factor analysis.^14^

After considering the scree plot and possible interpretations of the factors, we determined that the three factors were appropriate initial solutions. In deleting items after rotation, a factor loading criterion of 0.40 was used.^15^ Items that showed factor loadings of 0.30 or greater for multiple factors or items with factor loadings of less than 0.40 for all factors were deleted. Three factors were extracted after three rotations of repeated work to check for changes in the factor structure caused by item deletion. Inter-factor correlations ranged from 0.53 to 0.61 (Table 2). Correlations between subscales ranged from 0.50 to 0.54, and correlations between each subscale and the overall scale ranged from 0.81 to 0.84 (Table 3).

Factor 1 showed high factor loadings for seven items, including “Self-learn topics you have questions about” and “Take the initiative to work on your own”. These items were named *Positive actions toward self-reliance* because they comprised items that indicated actions toward independent nursing practice. Factor 2 showed high factor loadings for seven items, including “Ask about and confirm even minor details about how things are done at the current workplace” and “Notify staff that you require support, even if you have experience.” These items were named *Positive actions toward reliable nursing practice* because they indicated actions for careful, appropriate, and reliable nursing practice in the new workplace. Factor 3 showed high factor loadings for seven items, including “Familiarize yourself with the personalities of the staff through conversing with them” and “Showing humility.” These items were named *Positive actions toward building relationships* because they comprised items that indicated behaviors toward building smooth relationships. The Cronbach’s alpha coefficient for the overall scale was 0.89. On the subscale, *Positive actions toward self-reliance* scored 0.83, *Positive actions toward reliable nursing practice* scored 0.81, and *Positive actions toward building relationships* scored 0.79.

### Survey 2: Examining the reliability and validity of the Proactive Behavior Scale for Mid-Career Nurses

#### Background of participants

Responses were received from 225 participants (31.9% response rate). Of these, 50 participants with missing values and those who intended to retire were excluded, and 175 respondents were included in the analysis. Participants’ backgrounds are listed in Table 4.

#### Descriptive statistics

The median, minimum, and maximum values for the overall Proactive Behavior Scale for Mid-Career Nurses in the first survey were 4.19, 3.0, and 5.0, respectively; for the second survey, the median, minimum, and maximum values were 4.05, 3.0, and 5.0, respectively (Table 5).

#### Criterion-related validity

The Kolmogorov–Smirnov test confirmed that each scale followed a normal distribution. The Pearson’s product–moment correlation coefficient with the Scale of Proactive Behaviors in Organizational Socialization of New Graduate Nurses was 0.72 (*p*<0.01) for the overall scale, and the Pearson’s product–moment correlation coefficient for the Scale of Organizational Resocialization was 0.40 (*p*<0.01) for the overall scale (Table 6).

#### Test-retest reliability

The Kolmogorov–Smirnov test was used to confirm the normality of the first and second scores. The results confirmed that the first survey followed a normal distribution but not the second. The Spearman’s rank correlation coefficient was 0.75 (*p*<0.01) for the overall scale, 0.75 (*p*<0.01) for the subscale *Positive actions toward self-reliance*, 0.75 (*p*<0.01) for *Positive actions toward reliable nursing practice*, 0.66 (*p*<0.01) for *Positive actions toward building relationships*, and 0.70 (*p*<0.01) for positive correlation (Table 7).

## Discussion

### Examination of scale reliability and validity

#### Validity of extracted factors and adopted items

Factor 1, *Positive actions toward self-reliance*, extracted by the exploratory factor analysis, aggregated items indicating independent behavior in various aspects, including learning, nursing practice, and relationships with staff. Konishi identified learning issues for mid-career nurses in organizational socialization, including “acquiring the necessary knowledge and skills in a new department,” and “behaviors that contribute to the new department by using experience”.^8^ Factor 1 could be considered a behavior that contributes to the achievement of learning tasks, as described by Konishi. Furthermore, Ukawa conducted a conceptual analysis of proactive behavior and described three attributes of proactive behavior: “seeking unknown information,” “building relationships with others,” and “controlling one’s own cognition and making behavioral choices for adaptation.”^16^ Factor 1 of this scale included items that indicated behaviors of attempting to change one’s previous perceptions to fit the current workplace, as well as behaviors of attempting to learn or experiencing content that one lacks. These can be considered as behaviors aimed at cognitive adjustment and adaptation and were considered to correspond to the attribute “controlling one’s own cognition and making behavioral choices for adaptation,” as described by Ukawa.

Factor 2, *Positive actions toward reliable nursing practice*, comprised items that indicated behaviors to confirm or change methods acceptable in the current workplace rather than attempting to practice the methods of the previous workplace. These were considered to be contents that contribute to the achievement of the learning task described by Konishi, “attitude of acceptance of the norms and values of the new department”.^8^ In addition, Factor 2 included items that indicated behaviors to obtain new information needed to practice nursing in the current workplace and was considered to be commensurate with the attribute of proactive behavior described by Ukawa, “seeking unknown information”.^16^

Factor 3, *Positive actions toward building relationships*, comprised items indicating behaviors aimed at building smooth relationships. Behaviors toward building relationships have been shown to be proactive behaviors common to various industries, such as employees of general companies and new nurses,^6,9,17^ and similar results have been obtained for this scale. Furthermore, Factor 3 was also considered to correspond to the attribute “building relationships with others,”^16^ as described by Ukawa.

Thus, this scale was considered to retain content validity because it comprised items that indicate behaviors that contribute to the achievement of learning tasks in mid-career nurses’ organizational socialization. Furthermore, there were no differences in the conceptual structure of proactive behaviors, and construct validity was maintained.

#### Reliability study

The Cronbach’s alpha coefficient was 0.89 for the scale as a whole and 0.79 to 0.83 for the subscales; DeVellis found a Cronbach’s alpha coefficient of 0.65 to 0.70 to be a minimally acceptable range.^18^ Therefore, the scale was judged to have retained internal consistency.

The results of the test-retest method showed that Spearman’s rank correlation coefficient was 0.75 for the overall scale, indicating a strong correlation. The lower scales ranged from 0.66 to 0.75, with moderate to strong correlations.^19^ A measure is considered stable if its reliability coefficient is greater than 0.70.^13^ Thus, the scale was determined to be stable.

#### Criterion-related validity examination and characteristics of this scale

Pearson’s product–moment correlation coefficient between this scale and the Scale of Proactive Behaviors in Organizational Socialization of New Graduate Nurses score was 0.72, indicating a positive correlation between the two scales. Therefore, the scale retains its criterion-related validity. The finding of a correlation with the Scale of Proactive Behaviors in Organizational Socialization of New Graduate Nurses indicates that this scale comprises content that includes proactive behaviors common to both new graduate and mid-career nurses. In contrast, “Use your experience to find and implement possible contributions to your current workplace,” “Notify staff that you require support even if you have experience,” and “Accept the current workplace practices even if they vary from your previous workplace practices” were proactive behaviors unique to mid-career nurses, which were not observed in new graduate nurses.

The Pearson’s product–moment correlation coefficient between this scale score and the Scale of Organizational Resocialization score was 0.40, which was judged to ensure a certain degree of criterion-related validity. For this subscale, the correlation coefficient of Factor 1, *Positive actions toward self-reliance*, was 0.42, indicating a moderate level of correlation. As the Scale of Organizational Resocialization was developed based on a survey of sales employees, it was thought that Factor 1 comprised content that included proactive behaviors common to both general business employees and mid-career nurses. However, the correlation coefficient of this scale with Factor 2, *Positive actions toward reliable nursing practice*, was 0.21, indicating little correlation. One possible reason is that Factor 2 comprised items related to nursing practice, which differs from the proactive behaviors of employees in general companies. This suggests that Factor 2 of this scale indicates the proactive behavior characteristics of mid-career nurses.

### Potential of the scale

This scale is a simple and easy instrument with a short response time and low burden on users. The scale measures the content and extent of the proactive organizational adaptation behaviors that mid-career nurses engage in during organizational socialization. It is expected that mid-career nurses will be able to objectively view their behaviors toward organizational adaptation through self-evaluation using this scale, which will provide an opportunity for reflection. Organizations welcoming mid-career nurses can use the scale to understand the individual situation of mid-career nurses and consider support measures.

### Limitations and future implications

We have not yet confirmed the goodness-of fit of the factor models obtained from the exploratory factor analysis. Therefore, in future, it will be necessary to verify the goodness of fit between the three factors and the 21 items using the confirmatory factor analysis, and the utility of this scale should be further evaluated and revised.

## Conclusions

A Proactive Behavior Scale for Mid-Career Nurses was developed to measure their proactive behavior in the process of organizational socialization. This scale comprises 21 items with three factors: *Positive actions toward self-reliance*, *Positive actions toward reliable nursing practice*, and *Positive actions toward building relationships*. The reliability and validity of the scale were confirmed by examining its criterion-related validity, Cronbach’s alpha coefficient, and stability using the test-retest method.

## Figures and Tables

**Table1:** Background of participants of the survey to create the scale

		Number	%
Age	20s	64	17.4
	30s	135	36.8
	40s	116	31.6
	50s and over	52	14.2
Sex	Male	33	9
	Female	334	91
Total years of employment as a nurse	0～4 years	58	15.7
	5～14 years	162	44.2
	15 years over	145	39.5
	No answer	2	0.5
Number of beds (Current hospital)	20～99 beds	74	20.1
	100～299 beds	179	48.8
	300～499 beds	50	13.6
	500 beds over	60	16.3
	No answer	4	1
Department (Current hospital)	Internal Medicine	51	13.9
	Surgery	39	10.6
	Psychiatry	50	13.6
	Pediatrics	19	5.2
	Obstetrics and Gynecology	10	2.7
	Rehabilitation	28	7.6
	Mixed Departments	125	34.1
	Facilities for persons with severe motor and intellectual disabilities	9	2.5
	Long term care beds	7	1.9
	Regional Comprehensive Care Unit	5	1.4
	Palliative Care Unit	4	1.1
	Other	15	4.1
	No answer	5	1.4
Years of service (Current hospital)	Less than 1 year	85	23.2
	1～4 years	225	61.4
	5 years over	57	15.5

*N*=367

**Table2:** Exploratory factor analysis of the Proactive Behavior Scale for Mid-Career Nurses

Item		Factor loadings		
		Factor 1	Factor 2	Factor 3
**Factor 1 Positive actions toward self-reliance**				
Self-learn topics you have questions about.		**.73**	.05	.00
Prioritize learning by identifying the requirements of the current workplace.		**.68**	.03	.07
Before starting a new job, learn what you think you will need in the new workplace.		**.65**	–.08	–.05
Take the initiative to work on your own.		**.64**	.01	.03
Use your experience to find and implement possible contributions to your current workplace.		**.62**	.01	.08
Proactively and independently talk to staff members.		**.53**	–.09	.12
Notify staff that you want to practice what you lack experience with.		**.47**	.27	–.04
**Factor 2 Positive actions toward reliable nursing practices**				
Ask about and confirm even minor details about how things are done at your current workplace.		–.03	**.75**	–.04
Inform the staff member that you would like them to teach you how to do things at your current workplace despite your experience.		–.03	**.71**	.03
Inform staff members about your capabilities and limitations.		.21	**.69**	–.20
Notify staff that you require support even if you have experience.		–.21	**.66**	.16
If you are not confident in your ability to implement the program, communicate this openly to the staff.		.06	**.53**	.03
Accept the current workplace practices even if they vary from your previous workplace practices.		–.01	**.47**	.18
If you do not have any experience in the method, request a staff member to supervise you until you are familiar with the process.		.11	**.47**	–.04
**Factor 3 Positive actions toward building relationships**				
Familiarize yourself with the personalities of the staff through conversing with them.		.12	–.09	**.73**
Formulate an idea of a staff member’s personality and characteristics from conversations with other staff members.		–.03	.08	**.73**
Find a staff member who is easy to rely on.		–.05	.05	**.60**
Show humility.		.04	.06	**.60**
Interact with staff in similar contexts, such as mid-career employees or transfers within the hospital.		.08	–.17	**.48**
Act in alignment with the staff.		.02	.17	**.47**
Accept the attitude of the staff members, even if it is questionable.		–.01	.12	**.44**
Inter-factor correlation matrix	Factor1	—	.58	.53
	Factor2		—	.61
	Factor3			—
Cronbach’s alpha coefficient (Total=.89)		.83	.81	.79

*N*=367 Notes: Promax rotation by principal factor method; Numbers in bold are factor loadings .40 over Deletion criteria: items with factor loadings of less than .40 and items showing factor loadings of .30 over for multiple factors

**Table3:** Correlations among subscales of the Proactive Behavior Scale for Mid-Career Nurses

	Positive actions toward self-reliance	Positive actions toward reliable nursing practices	Positive actions toward building relationships	Total
Positive actions toward self-reliance	—	.53**	.50**	.84**
Positive actions toward reliable nursing practices		—	.54**	.82**
Positive actions toward building relationships			—	.81**

*N*=367 Note: Pearson’s product–moment correlation coefficient **: *p*<0.01

**Table4:** Background of participants of the study to examine the reliability and validity of the scale

		Number	%
Age	20s	34	19.5
	30s	53	30.3
	40s	55	31.4
	50s and over	32	18.3
	No answer	1	0.6
Sex	Male	16	9.1
	Female	158	90.3
	No answer	1	0.6
Total years of employment as a nurse	0～4 years	9	5.1
	5～14 years	82	46.8
	15 years over	83	47.4
	No answer	1	0.6
Number of beds (Current hospital)	20～99 beds	39	22.3
	100～299 beds	48	27.4
	300～499 beds	31	17.7
	500 beds over	40	22.9
	No answer	17	9.7
Department (Current hospital)	Internal Medicine	32	18.3
	Surgery	29	16.6
	Psychiatry	13	7.4
	Pediatrics	2	1.1
	Obstetrics and Gynecology	7	4
	Rehabilitation	9	5.1
	Mixed Departments	28	16
	Emergency	10	5.7
	Long term care beds	12	6.9
	Regional Comprehensive Care Unit	5	2.9
	Dialysis	3	1.7
	Other	10	5.7
	No answer	15	8.6
Years of service (Current hospital)	Less than 1 year	43	24.6
	1～4 years	100	57.1
	5 years over	30	17.1
	No answer	2	1.1

*N*=175

**Table5:** Descriptive statistics for the Proactive Behavior Scale for Mid-Career Nurses

		Median	Min	Max
Test 1	Total	4.19	3	5.00
	Positive actions toward self-reliance	4	2.43	5.00
	Positive actions toward reliable nursing practices	4.29	2.29	5.00
	Positive actions toward building relationships	4.29	2.71	5.00
Test 2	Total	4.05	3	5.00
	Positive actions toward self-reliance	4	2.57	5.00
	Positive actions toward reliable nursing practices	4.14	2.86	5.00
	Positive actions toward building relationships	4.14	2.86	5.00

*N*=175

**Table6:** Criterion-related validity results

		Scale of Proactive Behaviors in Organizational Socialization of New Graduate Nurses	Scale of Organizational Resocialization
		Total	Total
Proactive Behavior Scale in Organizational Socialization for Mid-Career Nurses	Total	.72**	.40**
	Positive actions toward self-reliance	.68**	.42**
	Positive actions toward reliable nursing practices	.54**	.21**
	Positive actions toward building relationships	.47**	.31**

*N*=175 Notes: Pearson’s product–moment correlation coefficient **: *p*<0.01

**Table7:** Results of the test-retest method

		Spearman’s rank correlation coefficient
Proactive Behavior Scale in Organizational Socialization for Mid-Career Nurses	Total	.75**
	Positive actions toward self-reliance	.75**
	Positive actions toward reliable nursing practices	.66**
	Positive actions toward building relationships	.70**

*N*=175 Notes: **: *p*<0.01

## Supplementary Material

PDF-Japanese
