# Supplementary material for: Development of the Proactive Behavior Scale for Mid-Career Nurses: a reliability and validity study
Source: Fujita Med J. 2024 Oct 31;11(1):20–7. doi: 10.20407/fmj.2024-015 (PMC11782940; doi:10.20407/fmj.2024-015)
Supplement: Supplementary file 1 — PDF-Japanese [file fmj-11-020-s001.pdf]

タイトル：

中途採用看護師のプロアクティブ行動尺度の開発―信頼性・妥当性の検討―

ランニングタイトル：

中途採用看護師のプロアクティブ行動尺度の開発

著者・所属：

Satomi Koyama, MSN[1,2], Yumiko Miyoshi, PhD[2], Kimie Takehara, PhD[2]

[1]Graduate School of Health Sciences, Fujita Health University, Toyoake, Aichi, Japan

[2]Faculty of Nursing, Fujita Health University, School of Health Sciences, Toyoake, Aichi, Japan

論文の種類：Original Article

Corresponding author：Kimie Takehara, PhD

連絡先住所：

Faculty of Nursing, Fujita Health University, School of Health Sciences, 1-98, Dengakugakubo, Kutsukake-cho, Toyoake, Aichi 470-1192, Japan

電話番号：0562-93-2554

メールアドレス：kimie.takehara@fujita-hu.ac.jp

アブストラクト

【目的】現在就業中の病院以外で、看護師として就業していた経験を有する看護師（中途採用看護師）が、組織社会化の過程で行っているプロアクティブ行動を測定する、中途採用看護師のプロアクティブ行動尺度を作成し、尺度の信頼性と妥当性を検討する。

【方法】中途採用看護師 875 名に中途採用看護師のプロアクティブ行動尺度案を用いた郵送調査を実施した。結果について探索的因子分析を行い、中途採用看護師のプロアクティブ行動尺度を作成した（調査 1）。さらに中途採用看護師 706 名に郵送調査を実施し、尺度の信頼性と妥当性を検討した（調査 2）。

【結果】探索的因子分析の結果から〔自立に向けた積極的行動〕、〔確実な看護実践に向けた行動〕、〔人間関係構築に向けた行動〕の 3 因子 21 項目からなる、中途採用看護師のプロアクティブ行動尺度を作成した。尺度全体の Cronbach の α 係数は 0.89、再テスト法の Spearman の順位相関係数は 0.75、外的基準である、新人看護師の組織社会化におけるプロアクティブ行動尺度と組織再社会化の尺度との Pearson の積率相関係数はそれぞれ 0.72、0.40 であった。

【結論】中途採用看護師のプロアクティブ行動尺度は信頼性と妥当性を保持していると判断できた。

キーワード：中途採用看護師、プロアクティブ行動、組織社会化、尺度開発

序論

質のよい看護を提供するためには、個々の看護師が自身の所属する病院組織の理念や価値観、行動様式等を認識し、それに基づいて看護を実践することが望まれる。看護師は離転職が多く、毎年 10 万人を超える看護師が再就業している[1-2]。病院ごとに組織文化や行動様式は異なり、看護実践方法にも違いがあると予測される。そのため、経験や実践能力があっても、中途採用看護師が再就職直後から新たな組織に合う考えや行動に基づいて看護を実践することは難しいと推察される。本来備わっている実践能力を十分に発揮できないことは、大きな損失といえる。したがって、看護の質を保証するためには、中途採用看護師の速やかな組織適応が重要と考える。

個人が新たな組織へ適応していく過程を組織社会化という[3]。組織社会化は学習の過程であり、組織の規範や行動様式を受け入れる文化的課題や、役割を遂行するための役割的課題、役割遂行に必要な技能を獲得するための技術的課題といった学習課題を達成することで促進していく[4-5]。この学習課題の達成は、中途採用看護師を迎える組織側からの支援と、中途採用看護師自身による取り組みとの両者によって促進していく。組織側からの支援は社会化戦術といわれ、OJT や研修などがある。一方、中途採用看護師自身による取り組みはプロアクティブ行動と捉えられる。小川はプロアクティブ行動を「組織内の役割を引き受けるのに必要な社会的知識や、技術を獲得しようとする個人の主体的な行動全般」と定義している[6]。プロアクティブ行動は社会化学習に影響を与えて組織社会化を促進させ、職場適応に至ることが明らかになっている[7]。小西は、中途採用看護師の組織社会化における学習課題とし

て「新規参入した部署で必要な知識・技術」、「新規参入した部署の規範や価値を受容する態度」、「経験を活かして新規参入した部署に貢献する行動」、「新規参入した組織の規範の理解」を明らかにしている[8]。したがって中途採用看護師の組織適応においては、これらの学習課題の達成に向けた、中途採用看護師自身によるプロアクティブ行動（組織社会化の過程で中途採用看護師自らが行う、組織適応に向けた主体的な行動）が重要といえる。

日本の看護領域におけるプロアクティブ行動研究は希少であり、唯一新人看護師を対象にした卯川の研究があるが、中途採用看護師については言及されていない[9]。そこで本研究では、中途採用看護師が組織社会化の過程で行っているプロアクティブ行動を測定する尺度の開発に着手した。尺度が開発できれば組織適応に有効なプロアクティブ行動が明らかになり、その行動を促す支援についても検討が可能になると考えられる。本研究を行うにあたり、これまでに中途採用看護師へのインタビューを基に作成した尺度の質問項目案の内容妥当性を確認し、60 項目からなる中途採用看護師のプロアクティブ行動尺度案を作成した。本研究の目的は、尺度案を用いて調査を行い、中途採用看護師のプロアクティブ行動尺度を作成することと、作成した尺度の信頼性と妥当性を検討することである。

方法

用語の操作的定義

1. *中途採用看護師*

本研究では、中途採用看護師を「現在就業中の病院以外で、看護師として就業していた経験を有する看護師」と定義する。

2.　プロアクティブ行動

本研究では、プロアクティブ行動を「組織社会化の過程で中途採用看護師自らが行う、組織適応に向けた主体的な行動」と定義する。

調査1　中途採用看護師のプロアクティブ行動尺度の作成

1.　対象者

各地方厚生（支）局のホームページに公開されている保健医療機関一覧を基に、全国にある 7,661 病院を 100 床ごとに層化した一覧表を作成した。調査票の目標回収者数を500名程度とし、協力が得られる病院の割合（30%程度とした）や調査票の回収率（30%程度とした）等を考慮して依頼する病院数を決定した。病床数で分類した一覧表ごとに乱数を生成し、比例層化無作為抽出法により調査依頼対象病院を選定した。これらの過程を経て、618 病院の看護責任者に調査を依頼し、承諾の得られた 97 病院に勤務する中途採用看護師 875 名を対象とした。

2.　調査内容

調査内容は、60 項目からなる中途採用看護師のプロアクティブ行動尺度案と基本属性であった。尺度項目の回答形式はリッカート法とし、各項目に対して7点「非常に当てはまる」から 1 点「全く当てはまらない」を配点した[10]。

3.　データ収集方法

2022年5月～8月に無記名自記式質問紙を用いた郵送調査を行った。協力が得られた病院の看護責任者宛てに、対象者数分の依頼文書および調査票、返信用封筒を送付し、対象者個々へ配布を依頼した。回収は、対象者が返信用封筒で個別に研究者に返送する方法を用いた。

4.　分析方法

欠損値と外れ値（上下ともに四分位範囲の1.5倍を超える値）を除外して分析対象者を決定した後に、各項目の得点分布を天井効果とフロア効果、尖度と歪度、ヒストグラムで確認した。さらに、項目間相関、修正済み項目合計相関（Corrected Item-Total Correlation：CITC）を確認した。項目分析を経たデータを用いて探索的因子分析を行い、尺度を作成した。確統計解析にはIBM SPSS Statistics 27を使用した。

調査2　基準関連妥当性と再テスト法による信頼性の検討

1.　対象者

調査1と同様に、自作の病院一覧から調査1とは異なる619病院を比例層化無作為抽出法により抽出し、看護責任者に調査を依頼した。そのうち承諾の得られた88病院に勤務する中途採用看護師706名を対象とした。

2.　調査内容

調査内容は、調査1を経て作成した中途採用看護師のプロアクティブ行動尺度と、新人看護師の組織社会化におけるプロアクティブ行動尺度、組織再社会化の尺度、基本属性であった。

1）　新人看護師の組織社会化におけるプロアクティブ行動尺度

卯川の新人看護師の組織社会におけるプロアクティブ行動尺度は、〔看護技術習熟行動〕5 項目、〔人間関係構築行動〕4 項目、〔積極的学習行動〕6 項目、〔他者からのフィードバック探索行動〕5 項目の 4 因子 20 項目から構成される 5 段階のリッカートスケールである [9]。この尺度は、新人看護師が組織社会化の過程で行っているプロアクティブ行動を測定するものであり、同じ看護職である中途採用看護師の組織社会化におけるプロアクティブ行動との関連が予測されるため、外的基準として採用した。

2）　組織再社会化の尺度

中原の組織再社会化の尺度は、〔人脈政治知識獲得〕7 項目、〔学習棄却〕4 項目、〔評価基準・役割獲得〕5 項目、〔スキル・知識獲得〕3 項目の 4 因子 19 項目から構成される 5 段階のリッカートスケールである [5]。この尺度は、中途採用を経験した営業職の社員を対象に行われた調査に基づいており、組織社会化の程度を測定するものである。プロアクティブ行動は組織社会化の促進要因とされていることから関連が予測されるため、外的基準として採用した。

3.　データ収集方法

2023 年 9 月～12 月に無記名自記式質問紙を用いた郵送調査を行った。尺度の安定性を確認するために、同一対象者に 2 週間から 1 ヶ月程度の間をあけて中途採用看護師のプロアクティブ行動尺度のみの調査を行った（再テスト法）。協力が得られた病院の看護責任者宛てに、対象者数分の依頼文書および 2 回分の調査票、返信用封筒を

送付し、対象者個々へ配布を依頼した。回収は、対象者が2回分の調査票をまとめて返信用封筒で個別に研究者に返送する方法を用いた。

4.　　分析方法

基準関連妥当性の検討では各尺度が正規分布であるか否かを確認した上で Pearson の積率係数を算出した。再テスト法による安定性の確認では、1回目の得点と 2回目の得点が正規分布であるか否かを確認した上で Spearman の順位相関係数を算出した。統計解析には IBM SPSS Statistics 27 を使用した。

倫理的配慮

対象者に対し研究の目的や方法、個人情報の保護、自由意思による研究参加等について文書で伝えた。研究協力の意思は質問紙調査票の返信をもって確認した。本研究は藤田医科大学医学研究倫理審査委員会の承認を受けて実施した（受付番号 HM20-609）。

結果

調査１　中途採用看護師のプロアクティブ行動尺度の作成

１）　対象者の背景

調査 1 は 506 名から回答を得た（回収率 57.8%）。そのうち、欠損値や外れ値等のある 32 名と、退職意向がある、または不明と回答した 107 名を除外したため、分析対象は 367 名であった。対象者の属性は表 1 の通りである。

2）　探索的因子分析

ヒストグラムにより、各項目の得点分布が単峰性であり大きな偏りがないことを確認した。尖度・歪度[11]、天井効果・フロア効果[12]、CITC[13]、項目が削除された場合の Cronbach の α 係数の確認を行い、基準を満たさなかった 11 項目を除外し、49 項目を探索的因子分析の対象とした。

主因子法、プロマックス回転を行った。Kaiser-Meyer-Olkin の標本妥当性は 0.94 であり、基準とされる 0.50 以上であった。Bartlett の球面性検定の p 値は 0.001 未満であり、共分散が 0 でなく、独立変数間に相関があることを示した。これらの結果から、因子分析をするにあたり問題のないデータであることを確認した[14]。

因子のスクリープロットおよび因子の解釈の可能性を考慮した結果、初期解としては 3 因子が妥当と判断した。回転後に項目を削除するにあたっては、因子負荷量の基準を 0.40 とした[15]。 複数因子で 0.30 以上の因子負荷量を示す項目や、すべての因子に対して因子負荷量が 0.40 未満である項目を削除した。項目の削除によって生じる因子構造の変化を確認する作業を繰り返し 3 回の回転を行ったところ、3 因子が抽出された。因子間相関は 0.53～0.61 の範囲であった（表 2）。下位尺度間相関は 0.50～0.54、各下位尺度と尺度全体の相関は 0.81～0.84 であった（表 3）。

第 1 因子は、「疑問に思ったことについて自己学習する」や「自分から率先して動く」など 7 項目が高い因子負荷量を示した。これらは自立した看護実践に向けた行動を示す項目で構成されていたことから〔自立に向けた積極的行動〕と命名した。第 2 因子は「些細なことでも現在の職場での方法を聞いて確認する」や「経験があって

も支援してほしいとスタッフに伝える」など7項目が高い因子負荷量を示した。これらは新たな職場における慎重かつ適切で確実な看護実践に向けた行動を示す項目で構成されていたことから〔確実な看護実践に向けた行動〕と命名した。第 3 因子は「スタッフとの会話を通してスタッフの人となりを知る」や「謙虚な姿勢を示す」など7項目が高い因子負荷量を示した。これらは円滑な人間関係の構築に向けた行動を示す項目で構成されていたことから〔人間関係構築に向けた行動〕と命名した。尺度全体の Cronbach の α 係数は 0.89 であった。下位尺度では、〔自立に向けた積極的行動〕が 0.83、〔確実な看護実践に向けた行動〕が 0.81、〔人間関係構築に向けた行動〕が 0.79 であった。

調査２　中途採用看護師のプロアクティブ行動尺度の信頼性と妥当性の検討

1）　対象者の背景

調査 2 は 225 名から回答を得た（回収率 31.9%）。そのうち欠損値および退職意向のある対象等 50 名を除外し、175 名を分析対象とした。対象者の属性は表 4 の通りである。

2）　記述統計

中途採用看護師のプロアクティブ行動尺度全体の 1 回目調査の中央値は 4.19、最小値は 3.0、最大値は 5.0 であった。2 回目調査の中央値は 4.05、最小値は 3.0、最大値は 5.0 であった（表 5）。

3）　基準関連妥当性

Kolmogorov-Smirnov の検定により、各尺度が正規分布に従うことを確認した。新

人看護師の組織社会化におけるプロアクティブ行動尺度との Pearson の積率係数は、尺度全体で 0.72（$p < 0.01$）、組織再社会化の尺度との Pearson の積率係数は、尺度全体で 0.40（$p < 0.01$）であった（表 6）。

4）　再テスト法による信頼性の検討

Kolmogorov-Smirnov の検定により、1 回目と 2 回目の得点の正規性を確認した。その結果、1 回目調査は正規分布に従うことが確認されたが、2 回目調査は確認されなかった。Spearman の順位相関係数は、尺度全体では 0.75（$p < 0.01$）、下位尺度では〔自立に向けた積極的行動〕が 0.75（$p < 0.01$）、〔確実な看護実践に向けた行動〕が 0.66（$p < 0.01$）、〔人間関係構築に向けた行動〕が 0.70（$p < 0.01$）であり、正の相関を認めた（表 7）。

考察

1.　本尺度の信頼性と妥当性の検討

1）　抽出された因子および採択された項目の妥当性

探索的因子分析により抽出された第 1 因子〔自立に向けた積極的行動〕には、学習面や看護実践、スタッフとの関係性など、様々な側面での自主的な行動を示す項目が集約された。小西は、中途採用看護師の組織社会化における学習課題とし「新規参入した部署で必要な知識・技術」や「経験を活かして新規参入した部署に貢献する行動」などを明らかにしている[8]。第 1 因子は小西が述べている学習課題の達成に寄与する行動と捉えることができた。さらに、卯川はプロアクティブ行動の概念分

析を行い「未知の情報を求める」、「他者との関係を構築する」、「自己の認知をコントロールし、適応のための行動選択をする」の3つをプロアクティブ行動の属性と述べている[16]。本尺度の第1因子には、これまでの認識を現在の職場に合わせて変化させようとする行動や、自分に不足している内容を学習したり経験したりしようとする行動を示す項目が含まれていた。これらは、認知の調整や適応に向けた行動と捉えることができ、卯川のいう属性「自己の認知をコントロールし、適応のための行動選択をする」に相応していると考えられた。

第2因子〔確実な看護実践に向けた行動〕には、以前の職場での方法をそのまま実践しようとするのではなく、現在の職場の方法を確認したり、現在の職場に受け入れられる方法へ変化させたりする行動を示す項目が集約された。これらは、小西が述べている学習課題「新規参入した部署の規範や価値を受容する態度」[8]の達成に寄与する内容であると考えられた。さらに、第2因子は現在の職場で看護を実践する上で、新たに必要になる情報を得ようとする行動を示す項目が含まれており、卯川のいうプロアクティブ行動の属性「未知の情報を求める」[16]にも相応していると考えられた。

第3因子〔人間関係構築に向けた行動〕は、円滑な人間関係の構築に向けた行動内容を示す項目が集約された。人間関係の構築に向けた行動は、一般企業の社員やや新人看護師など、様々な業種に共通するプロアクティブ行動であることが明らかになっており[6,9,17]、本尺度においても同様の結果が得られた。さらに、第3因子は、卯川のいう属性「他者との関係を構築する」[16]にも相応していると考えられた。

このように、本尺度は中途採用看護師の組織社会化における学習課題の達成に寄与する行動を示す項目で構成され、内容妥当性を保持していると考えられた。さらに、プロアクティブ行動の概念構造とも相違がなく、構成概念妥当を保持していると判断できた。

2）　信頼性の検討

本尺度の Cronbach の α 係数は、尺度全体では 0.89、下位尺度は 0.79～0.83 であった。DeVellis は Cronbach の α 係数について、0.65～0.70 は最小限許容できる範囲としている[18]。このことを考慮すると本尺度は内的一貫性を保持していると判断できた。

再テスト法の結果、Spearman の順位相関係数は、尺度全体では 0.75 であり、強い相関が認められた。下位尺度は 0.66～0.75 であり、中等度～強い相関が認められた[19]。通常、信頼性係数が 0.70 を上回れば、その測定法は安定しているとみなされる[13]。このことから、本尺度は安定した尺度であると判断できた。

3）　基準関連妥当性の検討と本尺度の特徴

本尺度得点と新人看護師の組織社会化におけるプロアクティブ行動尺度得点の Pearson の積率相関係数は尺度全体では 0.72 であり、両尺度間で正の相関が示された。したがって、本尺度は基準関連妥当性を保持していると判断できた。新人看護師の組織社会化におけるプロアクティブ行動尺度と相関が認められたことは、本尺度が新人看護師と中途採用看護師に共通するプロアクティブ行動を含む内容で構成されていることを意味する。一方で、「これまでの経験を活かし、現在の職場に貢献できることを見つけて実践する」や「経験があっても支援してほしいとスタッフに伝え

る」、「以前と異なっていても現在の職場の実践方法を受け入れる」などは、新人看護師には認められない中途採用看護師独自のプロアクティブ行動と考えられた。

本尺度得点と組織再社会化の尺度得点の Pearson の積率相関係数は尺度全体では 0.40 であり、一定程度基準関連妥当性を確保していると判断できた。下位尺度では、本尺度の第 1 因子〔自立に向けた積極的行動〕との相関係数は 0.42 であり、中程度の相関が認められた。組織再社会化の尺度は営業職の社員を対象とした調査に基づいて作成されていることから、第1因子は一般企業の社員と中途採用看護師に共通するプロアクティブ行動を含む内容で構成されていると考えられた。一方、本尺度の第 2 因子〔確実な看護実践に向けた行動〕との相関係数は 0.21 であり、ほとんど相関がみられなかった。その要因として、第2因子は看護実践に関する項目で構成されており、一般企業の社員のプロアクティブ行動とは異なることが考えられた。このことは、本尺度の第2因子が中途採用看護師に特徴的なプロアクティブ行動を示すものであることを示唆している。

2.　本尺度の活用可能性

本尺度は回答所要時間が短く簡便で使用者の負担が少ない尺度であると考えられる。尺度の測定結果は、中途採用看護師が組織社会化の過程で行っている、組織適応に向けた主体的な行動とその程度を示すものである。中途採用看護師は尺度を用いて自己評価することによって、組織適応に向けた自身の行動を客観視することができ、振り返りの機会になると期待できる。中途採用看護師を迎える組織側は、中

途採用看護師個々の状況を把握することや、支援策を検討する際に活用できると考えられる。

3.　本研究の限界と今後の課題

本研究の限界は、探索的因子分析の結果から得られた因子モデルの適合度について確認ができていないことである。確認的因子分析により、抽出された 3 因子と 21 項目の適合度について検証していく。さらに、尺度の実用性について評価と修正を重ねていく必要がある。

結論

中途採用看護師が組織社会化の過程で行っているプロアクティブ行動を測定するための中途採用看護師のプロアクティブ行動尺度を作成した。本尺度は〔自立に向けた積極的行動〕、〔確実な看護実践に向けた行動〕、〔人間関係構築に向けた行動〕の 3 因子 21 項目で構成される。基準関連妥当性、Cronbach の α 係数と再テスト法による安定性を検討した結果、尺度の信頼性と妥当性が確認された。

謝辞

本研究にご協力いただきました看護職員の皆様に深く感謝申し上げます。

利益相反

本研究における利益相反は存在しない。

引用・参考文献

1. Ministry of Health, Labour and Welfare. Kango shokuin no genjo to suii (Current Status and Transition of Nursing Staff); 2014 (in Japanese). <https://www.mhlw.go.jp/file/05-Shingikai-10801000-Iseikyoku-Soumuka/0000072895.pdf> (Accessed April 28, 2024)
2. Kobayashi M, Ikeda S. Kango shokuin no jukyu suikei no datosei to kyokyusu no shumireishon no kento (A study of the validity of nursing staff supply and demand estimates and simulation of supply numbers); 2022 (in Japanese) . <https://mhlw-grants.niph.go.jp/system/files/report_pdf/202022038A-buntan4.pdf> (Accessed June 3, 2024)
3. Van Maanen J, Schein EH. Toward a theory of organizational socialization. Research in Organizational Behavior 1979; 1: 209-64.
4. Katsuhara Y. Talking about my own nursing career. Tokyo: Shorinsha; 2024: 44-51 (in Japanese).
5. Nakahara J. Management Learning. Tokyo: University of Tokyo Press; 2012: 51-184 (in Japanese).
6. Ogawa N. Relative Importance of Organizational Socialization Tactics and Proactive Behaviors. Tokyo: The Research Institute for Innovation Management, Hosei University

2012: 1-40 (in Japanese).

7. Ashforth BE, Sluss DM, Saks AM.. Socialization tactics, proactive behavior, and newcomer learning: Integrating socialization models. Journal of Vocational Behavior 2007; 70: 447-62.
8. Konishi Y. Development of a Learning Scale for the Organizational Socialization of Reemployed Nurses. Osaka Prefecture University Doctoral dissertation 2021 (in Japanese).
9. Ukawa H, Hosoda Y. Development of a Scale of Proactive Behaviors in Organizational Socialization of New Graduate Nurses. Journal of Japan Academy of Nursing Science 2020; 40: 386-95 (in Japanese).
10. Oda K. A psychological study on the Japanese qualitative words . Japanese Journal of Educational Psychology 1970; 18: 166-76 (in Japanese).
11. Narita K, Shimonaka J, Nakazato K, Kawaai C, Sato S, Osada Y. A Japanese version of the generalized self-efficacy scale. Japanese Journal of Educational Psychology 1995; 43: 306-14 (in Japanese).
12. Oshio A. SPSS to Amos ni yoru sinri chosa deta kaiseki (Psychological and Survey Data Analysis with SPSS and Amos). Tokyo: TokyoTosho; 2004: 129 (in Japanese).
13. Polit DF, Beck CT. Nursing Research. 11th ed. Philadelphia: Wolters Kluwer; 2021: 350.
14. Tsushima E. SPSS de manabu iryokei tahenryo deta kaiseki (Medical Multivariate Data Analysis with SPSS). Tokyo: TokyoTosho; 2007: 145 (in Japanese).
15. Oshio A, Nishiguchi T. Shitsumonshi chosa no tejun (Questionnaire Survey Procedures).

Tokyo: Nakanishiya Shuppan; 2007: 101(in Japanese).

16. Ukawa H. Concept analysis of the proactive behaviors of newcomer: Application to new graduate nurses. International Nursing care Research 2018; 17: 27-33 (in Japanese).
17. Ashford SJ, Black JS. Proactivity during organizational entry: The role desire for control. Journal of Applied psychology 1996; 81: 199-214.
18. DeVellis RF, Thorpe CT. Scale Development. 5th ed. Los Angeles: Sage; 2022: 1-279.
19. Murakami Y. Shinri shakudo no tsukurikata (How to create a psychological scale). Kyoto: Kitaohji Shobo; 2006: 21 (in Japanese).

表 1. 尺度を作成するための調査対象者の属性

表 2. 中途採用看護師のプロアクティブ行動尺度の探索的因子分析

表 3. 中途採用看護師のプロアクティブ行動尺度の下位尺度間相関

表 4. 尺度の信頼性と妥当性を検討するための調査対象者の属性

表 5. 中途採用看護師のプロアクティブ行動尺度の記述統計

表 6. 基準関連妥当性の結果

表 7. 再テスト法の結果

表1. 尺度を作成するための調査対象者の属性

N = 367

		Number	%
Age	20s	64	17.4
	30s	135	36.8
	40s	116	31.6
	50s and over	52	14.2
Sex	Male	33	9
	Female	334	91
Total years of employment as a nurse	0～4 years	58	15.7
	5～14 years	162	44.2
	15 years over	145	39.5
	No answer	2	0.5
Number of beds (Current hospital)	20～99 beds	74	20.1
	100～299 beds	179	48.8
	300～499 beds	50	13.6
	500 beds over	60	16.3
	No answer	4	1
Department (Current hospital)	Internal Medicine	51	13.9
	Surgery	39	10.6
	Psychiatry	50	13.6
	Pediatrics	19	5.2
	Obstetrics and Gynecology	10	2.7
	Rehabilitation	28	7.6
	Mixed Departments	125	34.1
	Facilities for persons with severe motor and intellectual disabilities	9	2.5
	Long term care beds	7	1.9
	Regional Comprehensive Care Unit	5	1.4
	Palliative Care Unit	4	1.1
	Other	15	4.1
	No answer	5	1.4
Years of service (Current hospital)	Less than 1 year	85	23.2
	1～4 years	225	61.4
	5 years over	57	15.5

表2．中途採用看護師のプロアクティブ行動尺度の探索的因子分析

N = 367

Item	Factor loadings		
	Factor 1	Factor 2	Factor 3
**Factor 1 Positive actions toward self-reliance**			
Self-learn topics you have questions about.	**.73**	.05	.00
Prioritize learning by identifying the requirements of the current workplace.	**.68**	.03	.07
Before starting a new job, learn what you think you will need in the new workplace.	**.65**	-.08	-.05
Take the initiative to work on your own.	**.64**	.01	.03
Use your experience to find and implement possible contributions to your current workplace.	**.62**	.01	.08
Proactively and independently talk to staff members.	**.53**	-.09	.12
Notify staff that you want to practice what you lack experience with.	**.47**	.27	-.04
**Factor 2 Positive actions toward reliable nursing practices**			
Ask about and confirm even minor details about how things are done at your current workplace.	-.03	**.75**	-.04
Inform the staff member that you would like them to teach you how to do things at your current workplace despite your experience.	-.03	**.71**	.03
Inform staff members about your capabilities and limitations.	.21	**.69**	-.20
Notify staff that you require support even if you have experience.	-.21	**.66**	.16
If you are not confident in your ability to implement the program, communicate this openly to the staff.	.06	**.53**	.03
Accept the current workplace practices even if they vary from your previous workplace practices.	-.01	**.47**	.18
If you do not have any experience in the method, request a staff member to supervise you until you are familiar with the process.	.11	**.47**	-.04
**Factor 3 Positive actions toward building relationships**			
Familiarize yourself with the personalities of the staff through conversing with them.	.12	-.09	**.73**
Formulate an idea of a staff member's personality and characteristics from conversations with other staff members.	-.03	.08	**.73**
Find a staff member who is easy to rely on.	-.05	.05	**.60**
Show humility.	.04	.06	**.60**
Interact with staff in similar contexts, such as mid-career employees or transfers within the hospital.	.08	-.17	**.48**
Act in alignment with the staff.	.02	.17	**.47**
Accept the attitude of the staff members, even if it is questionable.	-.01	.12	**.44**
Inter-factor correlation matrix — Factor1	—	.58	.53
Factor2		—	.61
Factor3			—
Cronbach's alpha coefficient（Total＝.89）	.83	.81	.79

Notes: Promax rotation by principal factor method; Numbers in bold are factor loadings .40 over

Deletion criteria: items with factor loadings of less than .40 and items showing factor loadings of .30 over for multiple factors

表3. 中途採用看護師のプロアクティブ行動尺度の下位尺度間相関

N = 367

	Positive actions toward self-reliance	Positive actions toward reliable nursing practices	Positive actions toward building relationships	Total
Positive actions toward self-reliance	—	.53**	.50**	.84**
Positive actions toward reliable nursing practices		—	.54**	.82**
Positive actions toward building relationships			—	.81**

Note: Pearson's product–moment correlation coefficient **: $p < 0.01$

表4. 尺度の信頼性と妥当性を検討するための調査対象者の属性

N = 175

		Number	%
Age	20s	34	19.5
	30s	53	30.3
	40s	55	31.4
	50s and over	32	18.3
	No answer	1	0.6
Sex	Male	16	9.1
	Female	158	90.3
	No answer	1	0.6
Total years of employment as a nurse	0～4 years	9	5.1
	5～14 years	82	46.8
	15 years over	83	47.4
	No answer	1	0.6
Number of beds (Current hospital)	20～99 beds	39	22.3
	100～299 beds	48	27.4
	300～499 beds	31	17.7
	500 beds over	40	22.9
	No answer	17	9.7
Department (Current hospital)	Internal Medicine	32	18.3
	Surgery	29	16.6
	Psychiatry	13	7.4
	Pediatrics	2	1.1
	Obstetrics and Gynecology	7	4
	Rehabilitation	9	5.1
	Mixed Departments	28	16
	Emergency	10	5.7
	Long term care beds	12	6.9
	Regional Comprehensive Care Unit	5	2.9
	Dialysis	3	1.7
	Other	10	5.7
	No answer	15	8.6
Years of service (Current hospital)	Less than 1 year	43	24.6
	1～4 years	100	57.1
	5 years over	30	17.1
	No answer	2	1.1

表5.　中途採用看護師のプロアクティブ行動尺度の記述統計

N = 175

		Median	Min	Max
Test 1	Total	4.19	3	5.00
	Positive actions toward self-reliance	4	2.43	5.00
	Positive actions toward reliable nursing practices	4.29	2.29	5.00
	Positive actions toward building relationships	4.29	2.71	5.00
Test 2	Total	4.05	3	5.00
	Positive actions toward self-reliance	4	2.57	5.00
	Positive actions toward reliable nursing practices	4.14	2.86	5.00
	Positive actions toward building relationships	4.14	2.86	5.00

表6. 基準関連妥当性の結果

N = 175

		Scale of Proactive Behaviors in Organizational Socialization of New Graduate Nurses	Scale of Organizational Resocialization
		Total	Total
Proactive Behavior Scale in Organizational Socialization for Mid-Career Nurses	Total	.72**	.40**
	Positive actions toward self-reliance	.68**	.42**
	Positive actions toward reliable nursing practices	.54**	.21**
	Positive actions toward building relationships	.47**	.31**

Pearson's product–moment correlation coefficient　**：$p < 0.01$

表7. 再テスト法の結果

N = 175

		Spearman's rank correlation coefficient
Proactive Behavior Scale in Organizational Socialization for Mid-Career Nurses	Total	.75**
	Positive actions toward self-reliance	.75**
	Positive actions toward reliable nursing practices	.66**
	Positive actions toward building relationships	.70**

Notes: **：$p < 0.01$
